# Brainard District Medical Society

**Published:** 1880-04

**Authors:** J. A. Walker

**Affiliations:** Secy.


### Society Reports.

### ARTICLE X.

BRAINARD DISTRICT MEDICAL SOCIETY met in Mason City, Illinois, January 22, 1880. Present, Drs. H. B. Brown, C. H. Norred and R. M. Wilson, of Lincoln; Dr. S. T. Hurst, of Greenview; Dr. J. D. Whitley, of Petersburg; Dr. Ch. B. Maclay, of Delavan, and Drs. J. P. Walker, A. M. Bird, W. P. Walker, J. W. Spear and J. A. Walker, of Mason City. The president, Dr. Newcomer, being absent, Vice-President Whitley presided.

The constitution and by-laws, amended at a former meeting, were read by Dr. Wilson, chairman of committee, adopted and one hundred copies thereof ordered printed.

Dr. Whitley presented a supplementary report and photograph of a case of pleuritis requiring operation, reported at last meeting. Secretary instructed to forward Dr. Whitley's case and photo. to CHICAGO MEDICAL JOURNAL AND EXAMINER.

Dr. S. T. Hurst reported an interesting case of hydrocele, treated by tapping. On tapping the third time, found his hydrocele had become an hæmatocele. After evacuating, he thoroughly kneaded the scrotum, and sent the patient home. Complete recovery in three weeks, without any further treatment.

The afternoon session was devoted to the discussion of scarlatina, participated in by nearly all of those present. Dr. Whitley spoke very strongly in favor of the sulpho-carbolate of soda as a prophylactic; had seen very marked proofs of its value in his own practice.

Scarlatina will be the special subject for discussion at next meeting, April 22, 1880, at Mason City, Ill,

J. A. WALKER, *Secy.*

